# Gender-based generalisations in school nurses’ appraisals of and interventions addressing students’ mental health

**DOI:** 10.1186/s12913-016-1710-1

**Published:** 2016-08-30

**Authors:** Per-Åke Rosvall, Stefan Nilsson

**Affiliations:** 1 Department of Applied Educational Science, Umeå University, Per-Åke Rosvall, TUV plan 4, 90187 Umeå, Sweden; 2 Institute of Health Care Sciences, University of Gothenburg, Gothenburg, Sweden

**Keywords:** Gender, School nurses, Mental health, Secondary schools, Upper secondary schools

## Abstract

**Background:**

There has been an increase of reports describing mental health problems in adolescents, especially girls. School nurses play an important role in supporting young people with health problems. Few studies have considered how the nurses’ gender norms may influence their discussions.

**Methods:**

To investigate this issue, semi-structured interviews focusing on school nurses’ work with students who have mental health problems were conducted. Transcripts of interviews with Swedish school nurses (*n* = 15) from the Help overcoming pain early project (HOPE) were analysed using theories on gender as a theoretical framework and then organised into themes related to the school nurses’ provision of contact and intervention. The interviewees were all women, aged between 42–63 years, who had worked as nurses for 13–45 years, and as school nurses for 2–28 years. Five worked in upper secondary schools (for students aged 16–19) and 10 in secondary schools (for students aged 12–16).

**Results:**

The results show that school nurses more commonly associated mental health problems with girls. When the school nurses discussed students that were difficult to reach, boys in particular were mentioned. However, very few nurses mentioned specific intervention to address students’ mental health problems, and all of the mentioned interventions were focused on girls. Some of the school nurses reported that it was more difficult to initiate a health dialogue with boys, yet none of the nurses had organized interventions for the boys.

**Conclusions:**

We conclude that generalisations can sometimes be analytically helpful, facilitating, for instance, the identification of problems in school nurses’ work methods and interventions. However, the most important conclusion from our research, which applied a design that is not commonly used, is that more varied approaches, as well as a greater awareness of potential gender stereotype pitfalls, are necessary to meet the needs of diverse student groups.

**Electronic supplementary material:**

The online version of this article (doi:10.1186/s12913-016-1710-1) contains supplementary material, which is available to authorized users.

## Background

The prevalence of mental health problems among school children has increased [1] and how healthcare services are utilized is important to how problems are detected and how patients use those services [2]. According to Hagquist [3], mental health problems doubled among adolescent boys and tripled among adolescent girls in Sweden during the years 1988–2005. Moreover, research articles and reviews show that it is a pattern afflicting multiple countries [4, 5]. Frequently, the conditions are associated with recurring pain symptoms, such as headaches, stomach aches, and neck pain [6, 7]. In addition, a gender-based trend in reporting mental health problems has been noticed, as proportions of men and women who seek help for their problems differ [8, 9]. Furthermore, when afflicted by mental health problems, women more commonly experience feelings of helplessness to improve their conditions, phobias, panic attacks and/or free-floating anxiety states, while men more commonly exhibit an aggressive and antisocial character or substance dependence [9].

The reliability of some apparent differences between sexes in incidences of mental health problems may be questionable, as several studies have found that it is more socially acceptable for girls/women than for boys/men to admit to having mental health problems [10, 11] and that boys/men worry more about the impact of mental health-related stigma [12–14]. Thus, men’s mental health problems may be reported less frequently than women’s problems. However, other studies have concluded that the greater tendency for women to report mental health problems may also be partly due to the historically uneven distribution of power between genders, as women have traditionally had relatively weak control over various parts of their lives, less economic independence than men and limited influence outside of the domestic area [15]. Since the Second World War many countries, including Sweden, have seen an increase in women’s participation in the work force. This, combined with development of a state child care system, has decreased women’s dependence and may have eased the pressure of adhering to a traditional gender role. In addition, a review by Massey reports that currently, some women feel increased pressure because they are expected to be successful both at work and at home. In other words, men have not entered the domestic environment to the same extent as women have entered professional employment [16]. Furthermore, vocations that traditionally attract women (e.g. as a nurse, teacher, or nursery worker) require higher qualifications today than in the past, whereas vocations associated with men (e.g. as a mechanic, builder, or truck driver) still only require an upper secondary education [17]. Thus, girls may feel greater pressure to achieve good academic grades for advancement at an early age.

Schools inevitably play important roles (positive and/or negative) in students’ mental health [18], which intimately affects their academic performance [19], and school nurses can play key roles in identifying students’ problems [20, 21]. A nurse’s professional obligations include patient confidentiality and detachment from educational issues, both of which seem to be important for developing a communicative relationship with young adults. This type of relationship is vital, as it does not jeopardize a student’s education or their relationship with parents [22] which has been reported to be a barrier in contacts with mental health services [23]. The interaction between students and school health services has received limited attention from a qualitative perspective [12], but quantitative studies have noted certain patterns. For example, Clausson et al. [10] reported that girls seek treatment from health services for mental health problems more often than boys, but boys are more likely to seek treatment for physical injuries.

This article reports findings from the Help overcoming pain early project (HOPE). The overall objectives of HOPE were to determine school nurses’ and students’ perceptions of Swedish schools’ provisions of support for pupils afflicted by mental health and consequences of the arrangements. However, the specific aim of this article is to analyse the gender dimensions of school nurses’ responses to semi-structured interview questions concerning the provision of support for students with mental health problems. Furthermore, we wanted to investigate whether gender perceptions influenced school nurses’ interventions. We aimed to contribute knowledge regarding two research questions. First, do school nurses include gender dimensions into their discussions of students with mental health problems? If so, how does this influence their decision to intervene, and ways they initiate dialogue with a student who has a potential mental health problem?

To the best of our knowledge, most studies on gender are quantitative and few provide detailed, qualitative analyses of the generalisations, contradictions, and possible implications for boys and girls with mental health problems. This is also the case for evaluations of school health services. Therefore, the presented study provides important knowledge through methods, analyses, and theories seldom used in this research area.

## Methods

### Participants and procedures

Transcripts of semi-structured interviews with Swedish school nurses (*n* = 15) from the HOPE project were analysed in this study. The interviewees were all women, aged between 42–63 years, who had worked as nurses for 13–45 years, and as school nurses for 2–28 years. All of the interviews were transcribed word by word by a professional secretary. Five worked in upper secondary schools (for students aged 16–19) and 10 in secondary schools (for students aged 12–16). The school nurses were recruited consecutively by phone, during which an appointment was made, and the final group was selected so that there was a degree of geographical variation, as how school nurses work can differ between municipalities (i.e. economic, political, and structural reasons, as well as impacts based on municipality size). The selected school nurses also varied in educational background because qualified district, paediatric nurse, and school nurses were included, and these jobs differ based on the level of education required (Bachelor’s or Master’s degrees). Furthermore, there was variation in the type of school, community or private, at which the nurses were employed. Nurses from schools in the city and schools in more rural areas were selected so that there was also geographical variation within municipalities. Finally, the nurses varied in terms of whether they worked at a secondary school (for students aged 12–16) or an upper secondary school (for students aged 16–19). The school nurses represented 13 different schools. The interviews were conducted from January to November 2015. This study includes 15 school nurses selected by criterion sampling. The interviews focused on school nurses’ work with students who had mental health problems. Fifteen students with mental health problems were also interviewed. The interviews with both nurses and students have been analysed in another article [24], which focuses on schools’ organized support for students with recurrent pain (regardless of gender). However, the analyses in this article focus only on the interviews with school nurses in accordance with the specific aim stated above. The interviews were conducted by the authors, both of whom contributed to the interview guidelines. Both have extensive experience in qualitative interviewing and all of the concepts used were discussed thoroughly.

Since we were interested in our respondents’ views, we chose an inductive research approach [25, 26] with open interview questions, such as “How do you organise your work around students with mental health problems?”, followed by “Can you give a specific example of that work and who are involved?” (See Additional file 1) In other words, we applied a qualitative research approach to investigate what was happening at the school’s based on the nurses’ perspectives rather than asking a set of pre-determined questions that may not have been important to the participants [27, 28]. Therefore, no specific questions about gender were included in the interview guidelines and if an interviewed school nurse did not mention gender issues then the topic was not supposed to be discussed. However, all school nurses mentioned gender issues to some extent, some more explicitly than others. Thus, our approach was similar to what Charmaz [27] calls constructing grounded theory. That is, we tried to be as open as possible during the interviews, but remained aware throughout our data collection and analysis that our previous orientations towards sociological aspects of education, e.g. social background [29] and gender [15], co-construct our understandings [26].

The focus of the initial reading and coding of the transcripts from students (who are not in the scope of this articles aim) and school nurses was on the participants’ responses towards the organisation and structure of the school health services, including interventions and contact between students and nurses. The analyses resulted in two articles; one addresses the contradiction that students are more interested in having both individual help and group interventions as lessons and classes while the school nurses predominantly offer individual help with few exceptions [24] and the second highlights how a large portion of school nurses’ work is focused mainly on the adolescents’ micro- and mesosystems, whereas few strategies are conducted on either an exo- or macrosystem level [forthcoming]. However, we noted from the excerpts that all of the school nurses mentioned gender issues (despite a lack of focus on it during the interviews), which prompted an analysis of the gender dimension. Thus, the excerpts were coded and analysed using Connell’s [15] theories on gender as a theoretical framework, which is in line with the notion proposed by Charmaz that grounded theory is a method in process [27]. Connell defines gender as a “structure of social relations that centres on the reproductive arena, and the set of practices that bring reproductive distinctions between bodies into social processes” ([15] p. 11). Drawing on this definition, we were interested in whether school nurses make gender generalisations while organizing their work for students with mental health problems, and how their actions could materialise into (reproductive) social processes. With the framework in mind we analysed the transcripts again as openly as possible in order not to only include explicit talk about boys and girls. The first author completed this analytic procedure with the support of the second author and a project group consisting of colleagues and senior advisors that served to increase credibility. Thereafter, the authors discussed the categories until they arrived at a consensus. The qualitative approach with few interviews made it possible to in depth try to understand the school nurses’ talks with the concepts of Connell, and the perceptions of gender dimensions related to the provision of support for students with mental health problems. One of Connell’s concepts is hegemonic masculinity, which is a non-existent ideal, with context-dependent content, that is usually characterized by heterosexuality, authority, aggression and technical competence. All men and women in one way or another construct themselves in relation to hegemonic masculinity, which according to Connell is constituted in a gender order. The gender order implies the separation between men and women and different power relations. Interview excerpts with norms associated with those power relations in relation to mental health is what is analysed in this article.

In the Swedish school system education and health programs are often organized separately. Each municipality is responsible for its own school system and most have one authority in charge of education and another in charge of health services, which includes the management of school nurses. This structure can complicate collaboration between educational and health staff groups. However, since the early 2000s various national legislative acts have obliged schools to pay more attention to the prevention of health problems as well as the strengthening of connections between health and education [24]. Although municipalities may still differ on certain aspects of health services in school as in for example a use of a health dialogue, the legislation has increased standardization of school health services in Sweden [30]. Most school nurses in Swedish municipalities offer students the opportunity to have a personal health dialogue at two points during compulsory schooling and during their first year of upper secondary school. The aim of these dialogues is to discuss the situation at school, relationships, leisure activities, physical activities, eating habits, tobacco, alcohol and drug use, sexuality, and perceived health [31].

## Results and discussion

The results section is organised according to the research questions, except for a brief introductory part that presents findings in relation to the structure of Swedish healthcare services. The results then focus on gender, beginning with whether school nurses include gender dimensions in their discussions of students with mental health problems. Next, we discuss how this influences their decision to intervene, and finally, how school nurses initiate a dialogue with a student who has a potential mental health problem. Certain findings are discussed in the results section because they are closely related to a specific excerpt. Hence, we did not want to create too much distance between the excerpt and the presented finding.

### Students’ contact with nurses: health surveys and dialogues

All the school nurses in our study evaluated the health of students who made and kept an appointment by first using a standard survey, then engaged in a health dialogue, based on the students’ responses to the questions in the survey. Participation in the health dialogue was voluntary for the students, but was usually offered in a formal manner indicating that it was obligatory.

School nurse 12: We have tried different ways of getting students to attend a health dialogue. One year we allowed the students to say ‘yes’ or ‘no’ if they wanted to go. It was a catastrophe! Only a fifth of the students came. Now they have to actively say ‘no’ to avoid coming, and if they don’t come we invite them two or three times, and sometimes we have to almost drag them in from the corridor.

School nurse 3: It is an offer! But there are few [students] who don’t come. If they don’t, they usually come later, with an excuse that they want to have their height measured or something.

Initial answers from the school nurses revealed that they saw these health dialogues as an important way to make contact with students, both boys and girls. However, as shown below, the nurses found that certain groups of students were more reluctant to attend health dialogues than others. Furthermore, the nurses found that certain groups of students were more prone than others to report mental health problems, and their interventions targeting specific groups of students differed.

### Mental health problems were frequently associated with girls

The nurses commonly associated mental health problems with girls during the interviews, as in the following examples:

Interviewer: How common would you say that mental health problems from stress are?

School nurse 5: One out of ten I would say. And it is usually girls that have those problems.

School nurse 15: They are more common with stress, and related to pain among girls. We find that girls have more pressure on them and they put more pressure on themselves.

Furthermore, some school nurses reported that boys and girls visited the health services for different reasons:

School nurse 7: Boys come more often to have their height measured. Girls come for pain and mental health problems.

However, one school nurse said that she saw little difference in numbers of boys and girls visiting health services, but that girls often came singly while boys come in large groups (“herd”):

School nurse 1: At times there are only girls that come to talk about mental health and stress. But now and then boys come in almost herds to talk about stress related headaches and such. It is difficult to see any difference.

The implications (if any) of this gender-related difference in behaviour are impossible to tell from this comment, but may warrant further attention.

### Special interventions were organized, but only for girls

The school nurses were also asked if they collaborated with educational staff for special interventions, or to arrange thematic days. A few of the nurses mentioned that they had participated in special interventions or thematic work. One had implemented an annual intervention program:

School nurse 6: When it comes to preventive work I annually try to educate all the 8th grade girls using the DISA-program [DISA stands for Depression in Swedish Adolescents].

Another nurse reported that she had used the same program, but more occasionally. Our interviews revealed that school nurses work with students both individually and in group interventions. Their interventions seem to focus more on individual and psychological factors than on sociological factors, in accordance with previous findings regarding school nurses’ attitudes towards health promotion for adolescent girls [32]. The DISA-program, mentioned by school nurses in our study, was designed especially for adolescent girls, and has received mixed reviews. Kvist et al. [33] argue that the program is difficult to grasp and overly centred on the individual. Hence, it may not be useful for all students, and more focus on sociological aspects, such as conflict solving, could help students improve their conditions [34]. In addition, It is also reported [35] that after the program some girls said they felt stigmatized and that mental health problems are only associated with girls. In contrast, when the program was adapted for both girls and boys, as in one study by Garmy, some positive effects were noted [34]. The most interesting aspect of such interventions in the context of this article is their targeting of a gender-based group (girls), but it is noteworthy that the DISA program specifically mentioned by several school nurses interviewed in our study seems to be more effective when genders are not segregated.

The DISA-program was not the only intervention mentioned during the interviews with school nurses. One mentioned organizing dance groups for girls. Dancing has previously been shown to improve the self-rated health of adolescent girls [36], so there are strong grounds for arranging such activities. However, no corresponding activities for boys were mentioned.

### School nurses find it difficult to contact certain groups (of boys)

As mentioned above, all the students were offered an appointment with the school nurse for a health dialogue. When the nurses were asked whether it was more difficult to get particular students to come to their appointment than others, quite a few reported that boys presented more of a challenge than girls. One apparent reason for this was group pressure. This has been previously noticed in schools that offered programs that traditionally attract more boys than girls, such as a technology program [17]. A school nurse who worked at an upper secondary school that offered a technology program describes how group pressure seems to create problems:

School nurse 15: 95–97 percent come to the health dialogue. That is fantastic! But some students often go to the doctor anyway and think that they do not need another appointment. And some boys on the Technology program haven’t wanted to come, for some unknown reason. One starts saying ‘I won’t go!’, then another one does, and so on. It seems like a kind of group pressure or something.

Other school nurses reflected that it was difficult to get boys to come to the health dialogue due to constructions of masculinities:

School nurse 2: Boys are more, errr, you know, they are strong men already at this age [laughs] they don’t think it’s appropriate to go to the school nurse so to speak.

This opinion is in line with Connell’s [37] theory of hegemonic masculinities, which holds that all individuals have to relate to dominant conceptions of masculinity, which are in a state of constant flux, although some elements are relatively stable due to traditions and cultures in social interaction. The hegemonic masculinities are idealizations of what it means to be a successful man, associated with (for example) independence, manual work, and participation in sports. Mental health problems can be seen as threats to personal independence, and thus masculinity. The nurses described some of the boys at school as aggressive, difficult to reach, and refusing invitations from health care services:

School nurse 10: Then there’s this small group of boys that is kind of ‘out of the system’. They have low school attendance, stay at home, play computer games, or are at school but not in class. In those groups there are probably strong norms of not showing weakness and a need to be tough towards authorities such as teachers. Those boys are very difficult to reach.

School nurse 14: Most of the boys come to me to talk, either at the health dialogue appointment or spontaneously. But I have a feeling that the ones I don’t reach are most in need. They’re kind of alienated from school and society as a whole already as fourteen-year olds.

These excerpts illustrate school nurses’ perceptions that it was more difficult to persuade boys to attend a health dialogue. The nurses also described how boys and girls discuss different health issues when visiting them. For example, one school nurse mentioned that it could be more difficult to get boys to talk about their mental health:

School nurse 11: Girls can start to talk about their mental health immediately. Boys on the other hand, err, they come to me because of some physical pain, and it can take a while before it comes out that something else is bothering them.

This statement reflects findings by Claussons et. al. [10], that boys commonly seek help from school nurses for physical pain or injuries. This behaviour is also consistent with the theory of hegemonic masculinities [37], as consulting the school nurse because of physical injury does not jeopardize the idealization of masculinity, as the pain of the injury can be associated with an active lifestyle. Mental health problems, however, are associated with the opposite, showing dependence.

Although many of the school nurses commented that they had problems initiating a health dialogue with boys, none of them mentioned undertaking any type of intervention to improve the situation. However, one nurse did criticize the organization of the staff and the current educational system:

School nurse 5: I have worked as a nurse for a long time, but as a school nurse for only two years. And I think there’s very little systematic quality work at schools. For example, eighteen percent of students, mostly boys, may miss school once a week on average, and I can’t understand how this can be acceptable. Then the health team [head, school nurse, special needs teacher, and welfare officer] work on this one hour, once a week, and the rest of the time we are all spread out [in school].

Although the school nurses most often reported problems in getting some groups of boys to use the health services, one also mentioned that it was difficult to have a dialogue with some groups of girls, for example those with weight problems:

School nurse 4: Girls who are overweight. They can be difficult to get to come to an appointment. They seem to be afraid to have their weight measured and talk about their problems.

Although only one school nurse mentioned that girls may be difficult to get to an appointment, and she only mentioned one such group of girls, her comment highlights risks of drawing erroneously general conclusions regarding gender associations. Without this comment the nurses’ responses would have consistently indicated that only difficulties in initiating dialogue with boys needed to be addressed, and again the needs of an important group of students may have been neglected.

### Limitations

As in all qualitative studies with small samples, drawing general conclusion from the findings is potentially problematic. However, our results are corroborated by previous quantitative findings that students’ use of school health services is affected by gender [10, 12]. Since our results support the findings from previous quantitative studies and were analysed through recognised theories on gender, i.e. Connell and Massey, our qualitative results could well be transferable to other contexts. This study is, nevertheless, limited by its small sample size and the low variation of the participants in terms of contexts and cultures. Furthermore, the qualitative approach with semi-structured interviews applied in this study allowed the nurses to discuss what they felt were the most important parts of their work, without prompting by the interviewers, and provided unanticipated insights into their work with students experiencing mental health problems.

The credibility of qualitative studies is largely influenced by the researchers and how they produce and interpret data. The application of theories that are recognised and commonly used within the academic community helped us distance ourselves from the material [28].

One of the main strengths of applying open, semi-structured interviewing was that interviewees could raise questions that they felt were important. Hence, the focus on gender was initiated by the participants rather than the researchers. The participants raised issues of gender without being specifically asked about the topic. The use of open questions limits the risk for potential confirmation bias. This research approach produces a lot of transcribed material and limits the number of participants, yet reveals important knowledge about possible contradictions and paradoxes in the participants’ understandings of their work.

### Implications

This research identifies weaknesses and areas for future development in school health services in Sweden, and provides an approach that could be used to identify gender generalizations in other Swedish municipalities and potentially various countries. The main issue identified was that individual counselling and interventions targeting girls do not provide support to other groups of students who distance themselves from the health services. In accordance with other authors [34, 38], we recommend stronger collaboration between groups of staff (school administration, teachers, nurses, and special needs personnel). Furthermore, school health service systems could consider using new technologies to improve services for students, as proposed by Bartlett [39]. Finally, it would be useful to consider programs that provide help regardless of gender, such as the person-centred approach suggested by Ekman et al. [40] as an alternative to the DISA program.

## Conclusions

The presented analysis of school nurses’ interview responses identified a reproduction of gendered understanding of mental health problems among students, which they commonly associated with girls. Given statistics from surveys confirming that women are more prone to experience, or at least report, mental health problems [4, 5, 8] and indications of increased pressure on women to be successful both at home and work [11, 16], the nurses’ perception of girls more frequently experiencing mental health problems could simply reflect reality. However, detailed consideration of the school nurses’ responses and theories on gender suggests that this may be a simplistic interpretation. This study adds important knowledge since the presented analyses are difficult to perform within statistical and quantitative survey designs.

When the school nurses discussed students that were difficult to reach, boys in particular were mentioned. This has been confirmed by another qualitative study and a systematic review of literature [10, 12]. However, our findings showed that school nurses who had mentioned this difficulty in reaching certain students had not taken any action to address, or solve, the problem. It is outside the scope of our interviews, but since the school nurses mostly referred to individual student counselling in their offices [24] and said that some boys do not come to school at all, or are reluctant to come to the nurse’s office due to constructions of masculinity (as one school nurse said), a change in their traditional method of work may be necessary to reach this group of boys.

Very few nurses mentioned a specific intervention that was meant to address students’ mental health problems, and all of the mentioned interventions were focused on girls. This was interesting, as one preventative intervention approach, DISA, which was originally designed for girls has since also been applied to boys, see Garmy et al. [41]). None of the interviewed nurses had planned programs specifically for boys, although they said it was more difficult to get boys to talk to about health issues and might need help for mental health problems. Since this pattern was detected in analyses of the transcripts, the nurses were not asked in the interviews about why they (apparently) prioritised girls’ needs. However, their general responses indicated that they paid most attention to those in most need of their help, due to their heavy workloads. If so, the nurses’ prioritization, which may be influenced by gender stereotypes, may exacerbate problems associated with resource limitations and the organization of health services [23, 42].

We noticed several paradoxes in the nurses’ responses that have not been previously reported. For example, some boys did not seem to receive mental health counselling when they come to talk about physical pain, even if the nurses suspect that there is an underlying mental health problem. Thus, the nurses appear to reinforce general perceptions that girls experience mental health problems much more than boys, potentially leading to boys with mental health problems not getting the help they need, and that girls readily talk about their problems, potentially leading to certain groups of girls that do not follow this pattern, such as those with weight problems, without adequate help. We conclude that generalisations can sometimes be analytically helpful, facilitating (for instance) the identification of problems in school nurses’ practices and interventions, as demonstrated here. However, the most important conclusion from our research, which applied a design that is not commonly used, is that more varied approaches, as well as a greater awareness of potential gender stereotype pitfalls, are necessary to meet the needs of diverse student groups.

It should be noted that girls are overrepresented amongst those afflicted by mental health problems not only in school health services, but also in primary paediatric care [43] and hospitals [44]. However, a review of experimental studies of pain in children and adolescents found few significant differences between the genders [45]. Accordingly, further research is needed to support the school nurses who work with children.

## Additional file

Additional file 1: Interview guide school nurses. (DOCX 13 kb)

## Abbreviations

DISA: Depression in Swedish Adolescents
WHO: World health organization
