# Supplementary material for: Gender-based generalisations in school nurses’ appraisals of and interventions addressing students’ mental health
Source: BMC Health Serv Res. 2016 Aug 30;16(1):451. doi: 10.1186/s12913-016-1710-1 (PMC5006424; doi:10.1186/s12913-016-1710-1)
Supplement: Additional file 1: — Interview guide school nurses. (DOCX 13 kb) [file 12913_2016_1710_MOESM1_ESM.docx]

Supplementary file 1

Interview guide school nurses

**Background questions:**

Age (note gender, do not ask).

Education and special education.

Year as a school nurse and nurse.

**Questions:**

How do you organise your work around students with mental health problems?

- Can you give a specific example of that work and who are involved?
  - At the following occasions (if not answered above):
    - health dialogue
    - unplanned visits
    - school absence

In your work how do you acknowledge the following in relation to mental health?

- (difficulties in) learning
- medication
- (upper secondary school) thoughts of not finishing their education

Does what is stated before in this interview differ in terms of the patients social background?

If not answered above:

What strategies or treatment do you offer patients with mental health problems?

What resources (time, material, economy) do you have to support students with mental health problems?

What resources do you find lack of?

If you would make an intervention for this group of youth with mental health problems, what would that be?

In order to get thick descriptions ask questions as:

- Can you tell more about that?
- Can you give a specific example? (or another)
- Is it how you usually work/do?
